# Hierarchical structure guides rapid linguistic predictions during naturalistic listening

**DOI:** 10.1371/journal.pone.0207741

**Published:** 2019-01-16

**Authors:** Jonathan R. Brennan, John T. Hale

**Affiliations:** 1 Department of Linguistics, University of Michigan, Ann Arbor, MI, United States of America; 2 Department of Linguistics, Cornell University, Ithaca, NY, United States of America; 3 Department of Linguistics, University of Georgia, Athens, GA, United States of America; Utrecht University, NETHERLANDS

## Abstract

The grammar, or syntax, of human language is typically understood in terms of abstract hierarchical structures. However, theories of language processing that emphasize sequential information, not hierarchy, successfully model diverse phenomena. Recent work probing brain signals has shown mixed evidence for hierarchical information in some tasks. We ask whether sequential or hierarchical information guides the expectations that a human listener forms about a word’s part-of-speech when simply listening to every-day language. We compare the predictions of three computational models against electroencephalography signals recorded from human participants who listen passively to an audiobook story. We find that predictions based on hierarchical structure correlate with the human brain response above-and-beyond predictions based only on sequential information. This establishes a link between hierarchical linguistic structure and neural signals that generalizes across the range of syntactic structures found in every-day language.

## Introduction

The hierarchical syntax of human language sets it apart from other communicative and cognitive systems [1], yet there is significant debate about the role that this syntax plays in how the brain understands and produces language in real-time [2, 3, 4]. While neural data is consistent with brain systems that track hierarchical syntax rapidly and incrementally during listening [5, 6, 7, 8], studies that explicitly compare hierarchical syntax with alternatives that lack hierarchy and only encode sequential information show mixed evidence for syntax [9, 10, 11]. We contribute this debate by using electroencephalography (EEG) to test whether linguistic predictions reflect hierarchicy, not just linear sequences, even when participants do something as simple as listen to an audiobook story.

Human language syntax conforms to a formal class of languages that permit recursive self-embedding [12, 13] and laboratory experiments indicate that such structure is necessary to account for some aspects of real-time processing (e.g. [14]). But, day-to-day language-use need not use the most complex syntactic representations licensed by these formal systems [15, 16]. While conceding that hierarchical linguistic structure might condition the comprehension of certain special sentence styles, some theorists contend that much language processing may proceed based largely on the simplest and most frequent syntactic constructions. Applying heuristic “short-cuts” based on linear sequences alone [17] offers one answer to how upcoming linguistic input is processed rapidly and efficiently [18, 19].

The idea that language comprehension should be based on linear sequences alone, rather than on any sort of grammar, has a distinguished history. It appears prominently in the perceptual strategies proposed by Thomas Bever [20]; this included the famous Strategy D that assumes the sequence N-V-N corresponds to Agent-Action-Patient. This line continues up to the present day in the work of Christiansen and Chater [21] for whom word sequences, rather than grammatical analyses, are the central explanatory element in theories of linguistic performance. Indeed, eye-tracking data [9, 22], event-related potentials (ERPs) [10], and functional magnetic resonance imaging data (fMRI) [23, 24] indicate that readers and listeners form predictions about word categories, parts of speech like Noun and Verb, based on the immediately preceding linear sequence of words.

For example, Lopopolo and colleagues [24] report that expectations about a word’s part-of-speech, conditioned just by the two immediately preceding words, modulate the fMRI signal from the middle-temporal gyrus, the left superior frontal gyrus, and other regions. Frank et al. [10] report that the N400 event-related potential (ERP) is sensitive to such sequence-based expectations when participants listen to isolated sentences that are taken from literary sources. This component, which is traditionally associated with semantically unexpected stimuli [25, 26], does not show sensitivity to expectations based on hierarchical structure in their analysis.

In contrast to Frank et al.’s result, fMRI studies have revealed left temporal brain regions that are sensitive to linguistic predictions based on hierarchy above-and-beyond the linear sequence of words [11]. But, fMRI data are only indirectly linked to the millisecond-level dynamic brain states characteristic of language processing. Prior studies using electrocorticography (ECoG) [6] and Magnetoencephalography (MEG) [8, 27] that tie electrophysiological dynamics with hierarchical processing do not explicitly pit hierarchy against sequential models.

A strong test for the general role of hierarchical structure in language-use is whether it is required during every-day language tasks which feature a wide range of sentence types. We operationalize this by asking participants to simply listen passively to a 12 minute audiobook story, the first chapter of Alice’s Adventures in Wonderland, while we record brain activity with EEG. EEG is highly sensitive to linguistic expectations [25, 26, 28]. To examine the cognitive representations that guide these expectations, we leverage the computational construct of a *language model* which describes a probability distribution of word-sequences [10]. We construct three such probabilistic language models: two which condition probabilities solely on solely based on linear sequence information, and one which conditions probabilities based on the hierarchical structure assigned to a particular sequence of words. Each model quantifies the probability of an upcoming word’s part-of-speech. We use multiple regression to test which model of linguistic expectations best characterizes EEG brain activity.

To preview our results, we find that word-by-word expectations that are conditioned by hierarchical structure capture variance in the EEG data above-and-beyond expectations that are conditioned by word sequences alone.

## Materials and methods

### Audiobook stimulus

The stimulus was chapter one of Alice’s Advenctures in Wonderland as read by Kristen McQuillan and distributed by librivox.org. The audio was slowed by 20% using the pitch-preserving PSOLA algorithm to improve comprehensibility and was normalized to 70 dB SPL. An independent rater judged the digitally altered stimulus to sound natural and to be easier to understand than the original. The stimulus lasted 12.4 minutes. This same stimulus has been used in prior work [11, 29].

The stimulus chapter comprises 2,129 words in 84 sentences which are on average 25.8 words long (SD = 24.2). As part of our analysis, described below, these sentences are parsed using the Stanford Parser [30]. The resulting tree-structures indicate that the stimuli have a reasonable syntactic diversity: The stimuli average 2.31 clauses each and attest, for example, 153 different types of VP rules. For comparison, standard Penn Treebank training split is 950,028 words. If we kept seeing new VP types at the same rate in that corpus as we see in our 2,129-word text, we would expect to find 67,000 such rules. Instead, that corpus attests just 3,691 different types of VP rules. This indicates that our stimuli exercise many of the most typical VP types.

### Three models of incremental prediction

We quantify the expectation of a word *w* conditioned by a linguistic context *C* via *surprisal* [31]:

$$\begin{array}{c} surprisal\left(w\right)=-log_{2}\left(p\left(w|C\right)\right) \end{array}$$

This yields a value in bits corresponding to the amount of information conveyed by a word: unpredicted words convey more information and have higher surprisal values. For all models, we focus on the part of speech (POS) of *w* while varying the complexity of *C*.

We follow prior work by modeling POS surprisal here, which is termed “syntactic surprisal” by Roark et al. [32]. This choice permits a close comparison with prior work using sequence-based models [23, 24], and also hierarchical grammars [11, 32]. In separate work we examine lexical surprisal itself using a different class of models [29]. The present analysis relies on an assumption that comprehenders form expectations at the POS-level. This assumption is consistent with prior ERP research that has examined the brain response to violations of word-category expectations [33, 34, 35], but the degree of abstractness in natural language predictions remains a matter of debate (cf. [36]).

In an *Ngram* model, *C* is the POS of the linearly preceding words. We use the two preceding words, an un-lexicalized trigram model, which has successfully modeled language-related eye-movements, ERPs, and fMRI signals [9, 10, 24]: *C_Ngram_* = *w*_*i*−2_, *w*_*i*−1_. *Ngram* surprisal estimates come from a trigram language model estimated with OpenGRM with Witten-bell smoothing [37].

Following Frank and colleagues [10] we also derive surprisal values from a three-level simple recurrent neural network (*SRN*) which conditions probabilities on a weighted sum over the full preceding context [38]: *C_SRN_* = *w*_1_, *w*_2_ … *w*_*i*−1_. *SRN* surprisal estimates come from a three-layer network estimated with the rwthlm toolkit [39]. Network structure follows that used by Frank et al. (see also [40]): The network has 36 input and output nodes (one for each POS) and a 500 unit recurrently connected hidden layer consisting of sigmoid units. The network was trained using BPTT (batch size and learning rate set to software defaults); training was stopped when perplexity on the development set stopped improving.

Against these sequential models, we test for contributions from hierarchical structure using a broad-coverage un-lexicalized context-free grammar (*CFG*) [41]. While human languages are arguably more expressive than the Context Free languages [13], CFG-definable structures have been the flash-point for debate about the role of abstract hierarchy in every-day language processing [2]. Here, *C* is the set of grammatical structures that are compatible with the sequence of words preceding *w* [42]: *C_CFG_* = *structure*(*w*_1_, *w*_2_ … *w*_*i*−1_). *CFG* surprisal estimates come from the Stanford parser probabalistic context-free grammar [30] excluding terminal rules [32]. Surprisal values from this probabilistic context-free grammar were estimated using EarleyX [43, 44]. This is the same algorithm used in [31] and [45].

These three language models were chosen to hew closely to previous work comparing sequential with hierarchical accounts [9, 10, 11]. Both the *SRN* and *CFG* models use the entire prior context. The *SRN* is capable in principle of encoding arbitrary dependencies, but in practice such architectures are limited in their capacity to recover hierarchical dependencies such as subject-verb agreement [46, 47]. This is because the *SRN* enforces a recency bias which limits its capacity to capture long-distance dependencies [46]. This property, as well as the prominence of SRNs in prior work arguing against hierarchy in language-comprehension, supports their use as a conservative baseline against which to test the contributions of the explicitly hierarchical *CFG* model. Alternative neural network architectures such as those with memory gates may better capture context-free hierarchical structure [8, 48, 49, 50] and can be seen as mechanisms for approximating the function carried out by our *CFG* model. Thus, our aim is not to evaluate neural network architectures for sentence comprehension against others, but rather to evaluate the cognitive representations that are involved in comprehension, which may be recognized by some neural network architectures or by other means.

Because linguistic expectations are flexible and sensitive to genre [51], all models were trained on POS sequences returned by the Stanford parser applied to the entire story text. Examining the average number of bits used to encode an upcoming word’s POS is a measure of “linguistic accuracy” in the sense of [9]. This measure shows that the trained models capture properties of target text. There are 36 POS terms, so without any context this value is *log*_2_(36) = 5.17 bits. The three models each improve on this: The average bits per word (i.e. the average surprisal) for *Ngram* is 2.95, for *SRN* is 3.25 and for *CFG* is 3.69. In other words, context *C* carries an average of 2.22, 1.92 and 1.19 bits of information for each model, respectively. The differences in mean surprisal between each of the models is statistically reliable (*Ngram* < *CFG*: *t*(2149) = 16.3, *p* < 0.001, *SRN* < *CFG*: *t*(2149) = 10.0, *p* < 0.001, *Ngram* < *SRN*: *t*(2149) = 14.4, *p* < 0.001).

**Fig 1:**
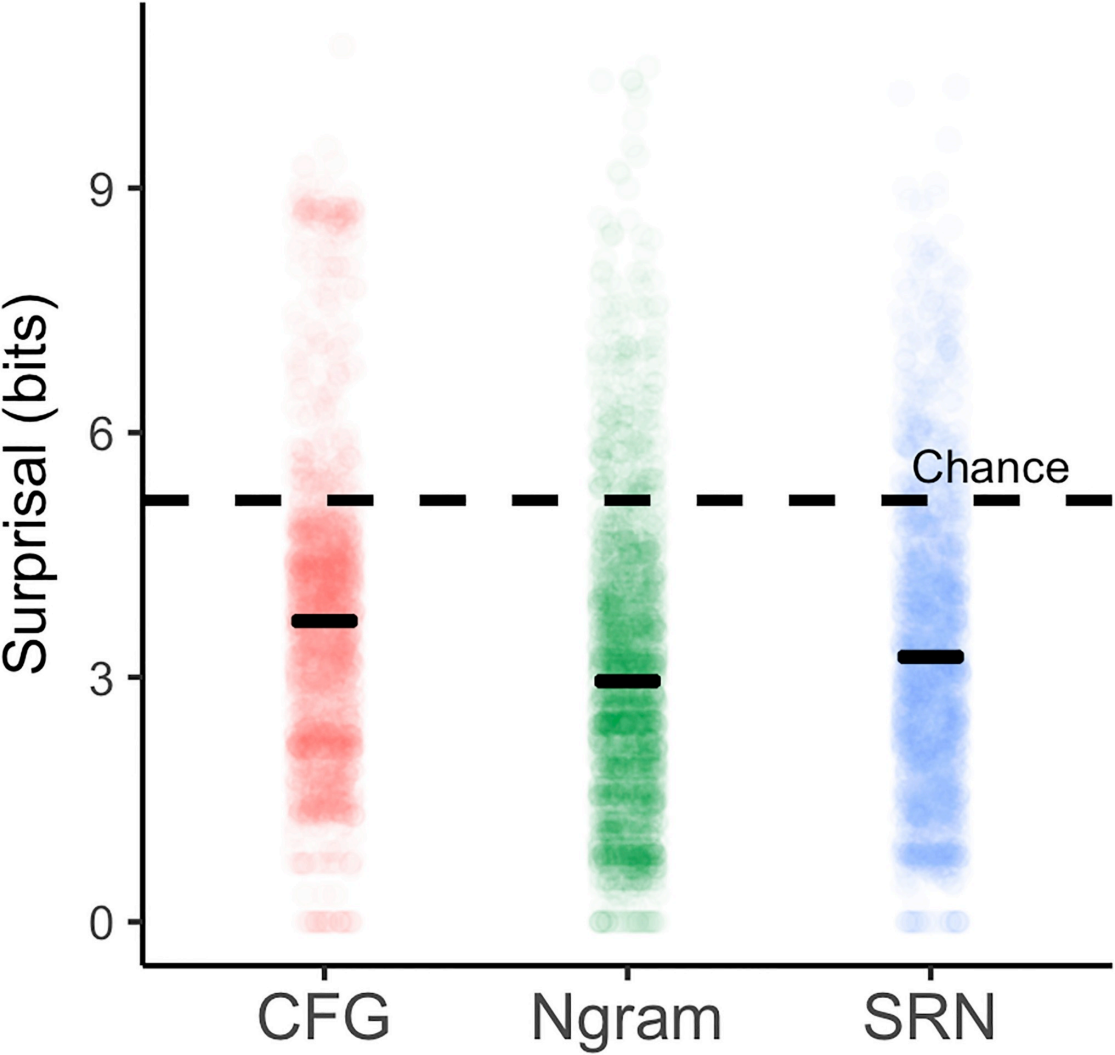
Surprisal distributions from each of three models along with mean surprisal ±1 standard error of the mean (black bars). Surprisal from a model where each POS tag is uniformly probably is indicated with the dashed line.

Fig 1 shows the distribution of surprisal values from the three models. These mean surprisal values are summarized in Table 1 along with the *perplexity* of each model, which is a common metric for comparing probability models (§8.3 [52]; see also S1 File). The perplexity of a model tested on words *w*_1_, *w*_2_…*w*_*N*_ is:

$$\begin{array}{c} 2^{-\frac{1}{N}\sum_{w=1}^{N}log_{2}\left(Pr\left(w|C\right)\right)} \end{array}$$

or, equivalently:

$$\begin{array}{c} 2^{mean(surprisal\left(w_{1},w_{2}\ldots w_{N}\right)} \end{array}$$

**Table 1:** Linguistic accuracy of language models. Model perplexity, average surprisal, and the bits of information encoded by the context in each of the three language models in comparison to a null model lacking context, where each POS tag is equally probable.

	Null	*Ngram*	*SRN*	*CFG*
Perplexity	36	7.74	9.52	12.90
Mean surprisal	5.17	2.95	3.25	3.69
Bits encoded in context	0	2.22	1.92	1.19

This analysis indicates that the models recover linguistically useful information and that sequential models do better than the hierarchical model in predicting an upcoming word’s POS.

**Fig 2:**
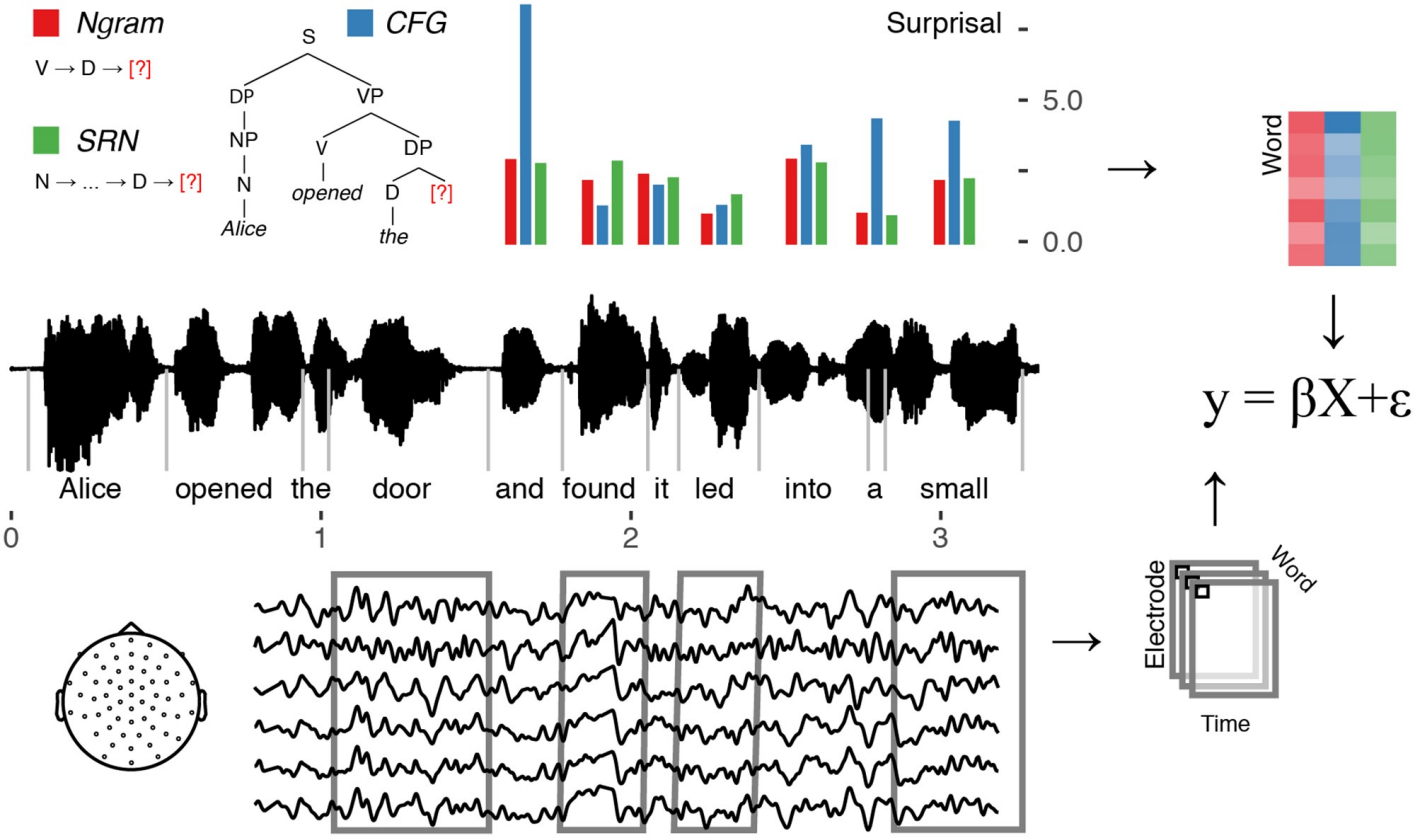
Language models and data analysis. Word-by-word surprisal values (top) estimated from one hierarchy-based and two sequential language models are time-aligned to the audiobook stimulus (middle). Epochs aligned with the onset of each word are extracted from filtered EEG data and amplitudes from each time-point and electrode serve as the dependent measure of a regression model (right) that includes surprisal values and low-level covariates as predictors.

Fig 2 (top) illustrates how word-by-word surprisal in a short passage of text may vary depending on whether the context used to condition those expectations includes sequential information (red and green bars) or includes hierarchical structure (blue bars) (see S1 File for more example sentences). Thus, while each model performs better than chance, they differ in how well they capture the expectation for each individual word. Following prior work [9, 10], we distinguish a model’s adequacy in capturing properties of the text with its adequacy in capturing human cognitive dynamics (“psychological accuracy”). The latter is our primary interest, which we address by testing how well the word-by-word predictions from these models match human sentence processing using electrophysiological signals that reflect linguistic expectations.

### Participants

52 adult volunteers, 44 women and 8 men, aged 18–28 (Median = 20), participated in the story-listening sessions. The volunteers provided written informed consent and were compensated $15/h for their participation. The experiment was approved by the University of Michigan Health Sciences and Behavioral Sciences Institutional Review Board (#HUM00081060).

An independent analysis of these data is reported by Hale et al. in a 2018 proceedings paper [29].

Data from three participants was not analyzed due to experimenter error and ten participants did not meet behavioral criteria (see below). Six datasets were excluded due to excessive noise during pre-processing, leaving 33 datasets for the final analysis. All exclusions were assessed prior to running the statistical analyses.

Participants completed an eight-question multiple choice questionnaire concerning the contents of the story at the end of the experimental session. Each question had four possible answers. Under the binomial distribution, correctly answering at least 5 questions is required to exceed chance at *α* = 0.05. We excluded data from all participants who did not meet this behavioral threshold. A supplemental analysis examines whether our results are sensitive to this behavioral exclusion criterion (S1 File).

### Procedure

After being briefed on the study procedure and providing informed consent, participants were fitted with an elastic cap with 61 actively-amplified electrodes and one ground electrode (actiCap, Brain Products GmbH). Electrodes were distributed equidistantly across the scalp according to the Easycap M10 layout. Conductive gel was inserted into each electrode to reduce impedences to 25 kOhms or below.

Participants listened to the stimulus with insert earphones (EA-2, Etymotic Inc.) in an isolated booth. Prior to hearing the audiobook, a hearing threshold was determined per participant and per ear using 1 kHz tones (300 ms, 10ms fade in/out). The audiobook story was played at 45 dB above this threshold.

Data were recorded at 500 Hz between 0.1 and 200 Hz referenced to an electrode placed on the right mastoid (actiCHamp, Brain Products GmbH). Following the 12.4 m story, participants completed an eight-question multiple-choice questionnaire asking about events in the story. The entire experimental session lasted 1–1.5 h.

### EEG data processing

Data processing was conducted using the Fieldtrip toolbox in MATLAB [53]. Raw EEG data were re-referenced to the average of left and right mastoid electrodes, high-pass filtered at 0.1 Hz, and divided into 2,129 epochs spanning -0.3–1 s around the onset of each word in the story (919 corresponding to content words, and 1,210 corresponding to function words). Ocular signals were removed using Independent Component Analysis [54] and remaining artifacts were identified and removed following visual inspection. 2.3%–26.2% of epochs were removed across participants (M = 13.5%), leaving on average 1,851 epochs for analysis per participant. Signals from electrodes with supra-threshold impedance or exceptional noise were replaced using surface spline interpolation [55] (Median = 4, Range = [0 12]). Each epoch was low-pass filtered at 40 Hz (4th order, butterworth). No baseline correction was applied.

### Statistical analysis

The statistical analysis addresses two questions: (i) what EEG signals reflect sentence-level surprisal, and (ii) which of these might reflect hierarchical structure above-and-beyond linear-sequence expectations. An initial whole-head EEG analysis identifies time-points and electrodes where EEG amplitudes are modulated by surprisal from any of the models we consider. A second ROI analysis uses step-wise model comparison to test for the unique contribution of each surprisal model.

#### Whole-head single-trial analysis

Single-trial linear regression was used per-participant at each time-point and electrode to identify EEG amplitudes that correlate with surprisal (Fig 2, right). Control predictors included *sentence order*, *word order* (within each sentence), *word frequency* (log-transformed) of the current, preceding, and following word, and *sound power* at word onset. *Word frequency* was based on the HAL corpus via the English Lexicon Project [56]. All predictors were mean-centered. Following [32], we conducted separate regression analyses for function words and for content words; a follow-up analysis explicitly tests for interactions with word-class. To these control predictors we added surprisal values from each of our three language models. We add these predictors in three separate regression models in order to best identify any candidate EEG signals that correlate with surprisal. A follow-up analysis using model comparison, described below, tests for the unique contribution of each language model above-and-beyond other terms.

Bivariate correlations between all predictors, prior to residualization, are shown in Table 2. We also constructed “null” regression models in which the rows of the design matrix were randomly permuted. These were used in the group-level analysis which is described next.

**Table 2:** Correlations between predictors. Bivariate correlation matrix giving Pearson’s *r* between terms entered into the single-subject regression models. sent: sentence order; word: word order within each sentence; frq, frq-, frq+: log-frequency for the current, previous, and following words; sndpwr: sound power at word onset; *Ngram, SRN, CFG*: surprisal from three language models. Off-diagonal correlations where |*r*| > 0.2 are highlighted with grey shading.

	**sent**	**word**	**frq**	**frq-**	**frq+**	**sndpwr**	***Ngram***	***SRN***	***CFG***
**sent**	1.00								
**word**	0.22	1.00							
**frq**	-0.02	-0.05	1.00						
**frq-**	-0.01	0.00	-0.15	1.00					
**frq+**	-0.02	-0.02	-0.15	0.00	1.00				
**sndpwr**	-0.01	-0.02	-0.01	-0.02	-0.01	1.00			
***Ngram***	-0.00	-0.06	-0.01	-0.15	-0.03	0.05	1.00		
***RNN***	0.00	-0.07	-0.03	-0.17	-0.01	0.05	0.84	1.00	
***CFG***	-0.00	-0.03	0.08	-0.05	-0.02	0.00	0.29	0.33	1.00

*β* coefficients for each effect were tested at the group level across time-points *t* from 0–1 s and across all electrodes *e* using a non-parametric permutation test [57]: (i) A dependent-samples *t*-test (df = 32) was conducted at each [*t*, *e*] comparing the target *β* against the matched *β* from the null model, (ii) [*t*, *e*] points with *p* < 0.05 were clustered based on spatio-temporal adjacency and their *t*-statistics were summed, (iii) Steps (i-ii) were repeated for 10,000 permutations where each single-subject regression result was re-asigned randomly to either the “target” or “null” conditions, and (iv) clusters with summed test statistics that exceeded at least 95% of the values from this permutation test were retained as “significant” at a multiple-comparison corrected *α* = 0.05.

#### Model comparison analysis

A second analysis used step-wise model comparison to test how well each surprisal model fits with the EEG data *above-and-beyond* the other models. These model comparisons were conducted over spatio-temporal regions of interest (ROI) that were defined by taking the union of all effects that were statistically significant according to the whole-head analysis, described above. By considering all sets of electrodes and time-points that were identified when each surprisal term was entered alone into a regression model, we minimize biasing this ROI analysis towards any one of our target models (c.f. [58]). In other words, each surprisal term was given equal footing to pick out electrodes and time-points at which it showed the strongest effect, and each such ROI was evaluated on equal grounds in these model comparisons.

**Fig 3:**
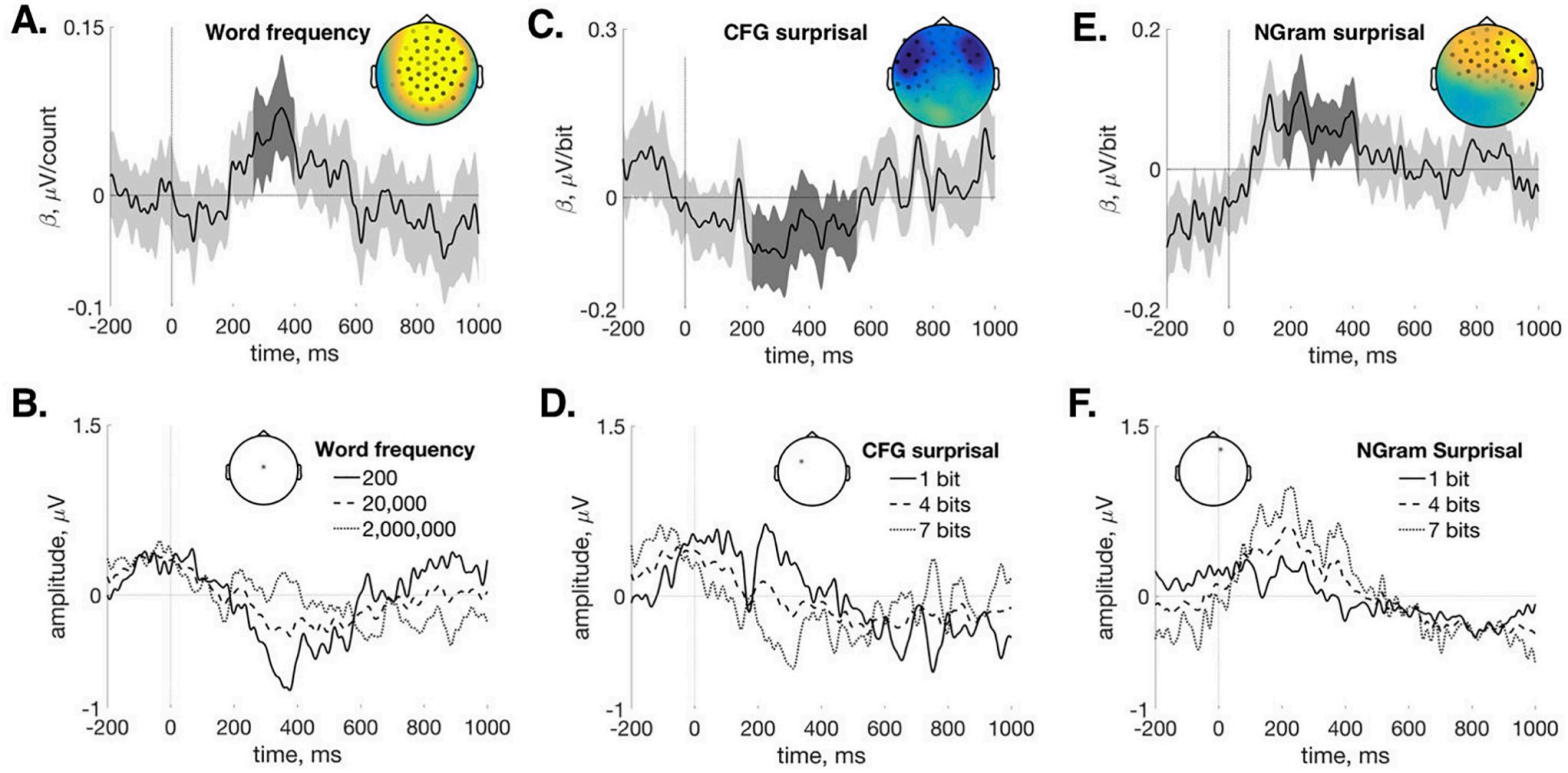
Whole-head regression results. *β* coefficients (*M* ± *CI*_95_) and model-reconstructed ERPs. (A) Regression time-series for log-transformed word frequency fit against content-word measurements. Dark grey shading indicates significant time-points and the inset shows significant channels and coefficient averages across significant time-points. (B) Estimated content-word ERP for three word frequency values ranging from low (200 corpus counts) to high (2,000,000 corpus counts) from a represenative central channel (inset) shows a classic N400 effect. (C) Regression time-series for hierarchical *CFG* surprisal fit against content-word data (inset and shading as in (A)). (D) Estimated content-word ERP for three *CFG* surprisal values from a representative channel (inset). (E) Regression time-series for sequential *Ngram* surprisal fit against function-word data (inset and shading as in (A)). (F) Estimated function-word ERP for three *Ngram* surprisal values from a representative channel (inset).

As described in the Results section, below, the whole-head analysis yielded five separate effects for *CFG*, *Ngram*, and *SRN*. Per-trial EEG amplitudes were averaged across the time-span of each effect from the five most statistically-robust electrodes. This was done separately for each effect even when there was overlap of electrodes and/or time-points to meet the goal of minimizing bias, as just mentioned. One of the effects had a strongly bimodel topography over left anterior and right anterior electrodes (see Fig 3C); we divided this effect into two ROIs across the midline. This procedure yielded six ROIs.

The step-wise comparisons were structured to test four statistical hypotheses based on our research question:

1. Does *CFG* improve model fits above-and-beyond both *Ngram* and *SRN*?
2. Does *Ngram* improve model fits above-and-beyond *CFG*?
3. Does *SRN* improve model fits above-and-beyond *CFG*?
4. Does *SRN* improve model fits above-and-beyond *Ngram*?

For each of these questions, we defined a baseline model which included all of the control predictors from the whole-head analysis described above, a binary term representing *word-class* (content vs. functional; sum-coded), and any surprisal terms that are being tested against (e.g. for point 1, above, this would be *Ngram* and *SRN*). For each such surprisal term, we also entered its interaction with word-class. Two types of model were compared against this baseline: A model in which we add just the surprisal term that we are testing for (e.g. *CFG* for point 1, above), and a model with that term and also its interaction with word-class.

The model comparison was conducted in R using the *brms* package [59] to construct fully Bayesian hierarchical regression models with the Stan programming language ([60]; see [61] for an introduction to Bayesian methods aimed towards language scientists). Models were fit with the brm() function containing population-level (“fixed”) terms and interactions as described above. All terms were centered and scaled so as to fall near to the range of ±10. Group-level (“random”) terms included by-subject intercepts and by-subject slopes for sentence-order and for word-class. Models were fit using four chains of 1000 warm-up iterations and 1000 sampling iterations. Prior distributions on all terms were the default values from brm(). Models were compared in terms of the Widely-Applied Information Criterion (WAIC; [62]) from the *loo* package. We highlight as “statistically significant” comparisons where the difference in WAIC exceeds two standard errors.

## Results

### Whole-head results

For content words, the word frequency control predictor correlates reliably with central activity in an interval spanning 266–400 ms, *p*_*mc*_ = 0.004. Fig 3A shows the grand-averaged regression coefficient time-course from electrodes that show this significant effect; this is an “rERP” plot following [63]. The predominant polarity during this time-span is negative-going. This is indicated in Fig 3B, in which the grand-average regression coefficients have been used to reconstruct an estimated ERP for words with different frequencies [63]. This result serves as a “sanity check”. It is consistent with the N400 ERP [64], replicating the familiar effect that more frequent words elicit smaller N400 amplitudes. This demonstrates that our analysis is sensitive to word-level variation within the naturally-presented story (see also [65]).

Hierarchical *CFG* surprisal correlates negatively with anterior activity from content words from 216–554 ms (*p*_*mc*_ < 0.001) (Fig 3C). The topography of this effect appears to be bi-modal over left-anterior and right-anterior electrodes. Averaging the regression coefficients across participants shows the model-estimated ERP for words with different *CFG* surprisal values at a left anterior electrode (Fig 3D).

There are no effects of *Ngram* or *SRN* surprisal on content-word activity (*Ngram* min(*p*_*mc*_) = 0.098; *SRN* min(*p*_*mc*_) = 0.378).

When considering grammatical function words, *Ngram*-based surprisal correlates positively with anterior activity in two time-windows: 102–158 ms and 174–420 ms (*p*_*mc*_ = 0.035 and *p*_*mc*_ = 0.001, respectively). The latter more right-lateralized of these is shown in Fig 3E. Both *SRN* and *CFG*-based surprisal show positive correlations with function-word activity that are very similar in topography and latency to the latter *Ngram* effect (*SRN*: 174-252 ms, *p*_*mc*_ = 0.011; *CFG*: 210–310 ms, *p*_*mc*_ = 0.021). The rERP plots for all significant effects are shown in S1 File.

### Model comparison results

**Fig 4:**
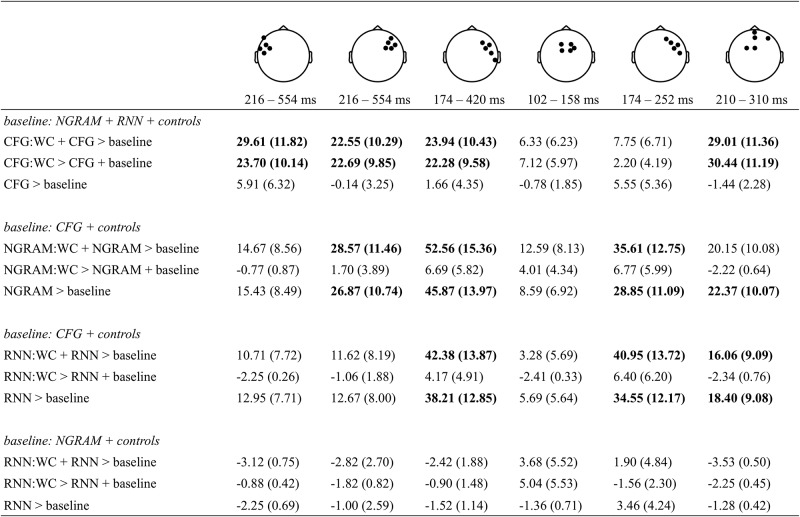
Model comparison results. WAIC difference scores (± standard error) indicate changes in model fit across six ROIs (columns). Each set of rows tests a different statistical question using step-wise model comparison. Terms that are being evaluated are indicated to the left of “>”; interactions with word-class are indicated with “:WC”. For each row-set, the baseline model includes all control covariates along with the indicated surprisal term(s) and interactions between word-class and those surprisal terms. The WAIC values are scaled so that positive numbers represent improvements for the larger model, while negative numbers indicate that the added complexity of the larger model is not matched by a better fit. Bold-face indicates WAIC improvements that are more than two standard errors from zero.

Step-wise model comparison tests for contributions of the hierarchical and sequential models *above-and-beyond* each-other and other control covariates. These comparisons also explicitly test for interactions with content or function word-class. Model-comparisons were tested in six spatio-temporal ROIs that were determined by the results of the whole-head analysis. Each of these ROIs are shown in the top-row of Fig 4. The remaining rows of Fig 4 appear in four sets, each of which corresponds to a specific statistical question concerning whether a target predictor significantly improves model fit above-and-beyond a model that includes other surprisal terms and covariates.

The top row-set tests whether *CFG*-based surprisal improves model fits above-and-beyond the fit achieved by a model already containing *Ngram*, *SRN*, interactions between both of those terms and word-class, and other control covariates. The results indicate just such an improvement. This is shown by the change in WAIC, a measure of model fit, that is indicated bold-face in four of six ROIs spanning left- and right-anterior electrodes from about 200 to 500 ms. These ROIs include those that were identified with the *CFG* term in the whole-head analysis (see Fig 3C), but crucially they also include ROIs defined with other terms, such as *Ngram* in the third column (see Fig 3E). This effect is not found in two ROIs spanning midline and right-lateralized electrodes and only early time-points (≈ 100–250 ms). Fit improvements are seen when *CFG* interacts with word-class, but not when *CFG* alone is tested against a baseline model. This indicates that *CFG*-based surprisal improves the fit against anterior scalp voltages in a window spanning roughly 200-500 ms, and this effect is specific to the EEG response to content-words.

The second and third row-sets in Fig 4 indicate that both *Ngram* and *SRN* improve model fits above-and-beyond *CFG* in several of the ROIs. This improvement is observed in right-anterior and midline electrodes in an interval spanning roughly 200 to 400 ms. Furthmore, improvement is seen when either the main effect of *Ngram* (second row-set) or *SRN* (third row-set) is compared to a baseline model. There is no reliable improvement in model fits when either surprisal term interacts with word-class. These results indicate that some aspects of the EEG record reflect sequential information independent of hierarchy, and such effects do not appear to be specific to content or to function words.

Of the two sequence-based models, there is no evidence that *SRN* shows improved fits compared to *Ngram* (Fig 4, fourth row-set). This may not be surprising given the similarity between the *Ngram* and *SRN* predictors (*r* > 0.8, see Table 2), and it is also consistent with the similar pattern of fits observed between the second and third row-sets in Fig 4.

Based on a comment from a Reviewer, We repeated the same set of step-wise model comparisons adding in data from participants who did not meet our behavioral criteria. The same qualitative patterns were observed with the larger sample of N = 41 for each of the four statistical questions. These model comparison results are reported in the supporting information (S1 File).

Diagnostic checks did not reveal any problems with the models used in this comparison. The residuals from each model were normally distributed with a first-order autocorrelation < 0.2; all terms had a R-hat value < 1.1, indicating that the Markov chains used in the estimation procedure were consistent (see https://github.com/stan-dev/stan/wiki/Stan-Best-Practices). Goodness-of-fit summaries are given in S1 File.

## Discussion

EEG signals collected during a natural story-listening task indicate that expectations do reflect the hierarchical structure of language above-and-beyond linear sequence information as represented in the control models used here. Negative voltages over frontal electrodes correlate with higher hierarchical surprisal for a word’s POS. These results are observed for content words beginning around 200 ms after the onset of a word, but not for function words. This result is consistent with prior research showing increased anterior negativities for unexpected word-categories (e.g. [33, 34, 66]) and suggests that these expectations are conditioned in part by hierarchical syntactic structures across a range of sentence types. The *CFG* surprisal results are robust against alternative model paramaterizations, such as whether or not variance associated with lower-order sequential surprisals are partialed out (Fig 4, row-set 1), or whether data from participants who did not meet behavioral criteria are factored in (S1 File).

The model-comparison results in the first row-set of Fig 4 indicate that *CFG* surprisal interacts with word-class such that scalp voltages are more negative for high surprisal content-word POS, but not for function words. There are several possible interpretations of this interaction. Psycholinguists have long recognized that unexpected content-words and unexpected function-words may lead to different processing strategies [67]. For example, unexpected content words may lead to more lexical access effort on the part of the comprehender, while unexpected function words may lead to increased effort in syntactic reanalysis. Or, when considering just POS, an unexpected adjective can be accomodated via adjunction to a noun phrase, while an unexpected auxilliary, determiner, or preposition, may require a more radical adjustment to one’s incremental interpretation. Such strategic differences are evident, for example, in the eye-tracking record where function words are much more commonly skipped, as compared to content words. This difference motivates [32] to completely separate these two word classes in their analysis of the effects of surprisal on eye-movements. The present result thus reinforces prior observations that the processing consequences of a violated expectation varies across different types of words, although these results do not alone narrow down how such processes might differ.

This *CFG* effect appears in both left-anterior and right-anterior electrodes after around 200 ms. The current analysis does not indicate whether the time-course of the effect may be different across the two hemispheres. The anterior topography contrasts with that of the N400 ERP, which is typically central or central-parietal [25, 26, 28]. But, the topography and time-course is similar to other anterior negativities, like the “left anterior negativity” LAN that has been associated with unexpected morphosyntax during language comprehension (e.g. [68], see also [69, 70]). In fact, a “early left anterior negativity” (ELAN; [34, 35, 66]), which overlaps with the *CFG* effect observed here, has been reported specifically for unexpected word-categories where context provides strong constraint (but see also [71] for a critical perspective).

In a report using the same dataset as studied here, Hale and colleagues [29] find an anterior positivity for high surprisal words in roughly the same time-window. That analysis is based an a different computational model which, crucially, characterizes surprisal for individual lexical items, not POS. It is possible that the differences in polarity between these two results may suggest a sensitivity to POS surprisal versus lexical surprisal. These comparisons invite further study in order to tease out the function(s) reflected by the anterior effects found in the present study. But we are cautious to speculate furthur here so as to avoid a “reverse inference” from the scalp topography to a specific functional interpretation.

The improved fit against EEG signals afforded by *CFG* surprisals contrasts with how well the three language models predict the text itself. The *CFG* model performs numerically worse than the two sequential models in terms of predicting a word’s POS based on the left context (Table 1). This observation militates against an alternative explanation for our findings: That the *CFG* results follow from “building in” additional sequence-based information via the grammatical rules. Such information could have, arguably, not been recovered by the *Ngram* or *SRN* models during training. However, the two sequential models perform better at the task of predicting upcoming POS terms; they do not suffer from a lack of training data. Still, the present experiment does not indicate the degree to which training effects (size of training data, genre, etc.) may impact the relative fit of alternative models; we aim to quantify the choice of training data in future work. Keeping this limitation in mind, we contend that the improved fits shown by the *CFG* model against EEG data reflect the fact that this model better matches the underlying representations used by the human participants during the task.

The present conclusions depend on taking the two sequence-based models tested as reasonable estimators of human-like sequence processing. Of course, these models are imperfect and noisy approximations of human processing; whether more complex sequence-based models might better capture latent hierarchical structure is a matter of ongoing research [47, 72]. But, the high performance of the current sequence-based models just noted, and their use in prior ERP studies of surprisal [10], recommend them as useful baseline models for this study.

In fact, statistically reliable effects for sequence-based surprisal from the *Ngram* and *SRN* models are also observed. *Ngram* and *SRN* surprisal correlates positively with right-frontal voltages in an early window beginning around 100 ms. In contrast to *CFG*, these sequence-based effects do not interact with word-class. This pattern does not correspond to a known language-related ERP component, but the timing and temporal topography is consistent with top-down modulation of sensory responses to language [35, 73, 74]. This effect holds even when when variance associated with *CFG* surprisal is first partialed out (Fig 4, row-sets 2 and 3, columns 3 and 5). We observe no effects for *SRN* surprisal when *Ngram* effects are partialed out (Fig 4, row-set 4). These results are consistent with some processes that reflect sequence-based expectations, and not just hierarchy [9, 10, 22, 24].

To conclude, hierarchical structure appears to condition word-by-word expectations even when participants perform a simple and natural task like listening to an audiobook. Hierarchy-based estimates for an upcoming word’s part-of-speech correlate with EEG-recorded scalp voltages in a time-window consistent with word-expectation effects from less natural laboratory tasks. These correlations are observed above-and-beyond estimates from sequential language models that have had prior success in fitting human reading times and ERPs. The present results generalize across a wide range of sentence types that appear in every-day language.

## Supporting information

S1 File Supporting figures & tables. Supplemental materials include further examples of model output, figures for all whole-head results, and analyses that include participants who did not meet behavioral criteria.
(PDF)

## References

[1] Hauser MD, Chomsky N, Fitch WT. The Faculty of Language: What is it, Who has it, and How did it Evolve? Science. 2002;298:1569–1579. 10.1126/science.298.5598.1569 12446899

[2] Frank SL, Bod R, Christiansen MH. How hierarchical is language use? Proceedings of the Royal Society B: Biological Sciences. 2012;. 10.1098/rspb.2012.1741 22977157 PMC3479729

[3] Ding N, Melloni L, Tian X, Poeppel D. Rule-based and word-level statistics-based processing of language: insights from neuroscience. Language, Cognition and Neuroscience. 2017;32(5):570–575. 10.1080/23273798.2016.1215477 29399592 PMC5794029

[4] Frank SL, Christiansen MH. Hierarchical and sequential processing of language. Language, Cognition and Neuroscience. 2018; p. 1–6.

[5] Ding N, Melloni L, Zhang H, Tian X, Poeppel D. Cortical tracking of hierarchical linguistic structures in connected speech. Nat Neurosci. 2016;19(1):158–64. 10.1038/nn.4186 26642090 PMC4809195

[6] Nelson MJ, El Karoui I, Giber K, Yang X, Cohen L, Koopman H, et al Neurophysiological dynamics of phrase-structure building during sentence processing. Proc Natl Acad Sci U S A. 2017;. 10.1073/pnas.1701590114 PMC5422821 28416691

[7] Henderson JM, Choi W, Lowder MW, Ferreira F. Language structure in the brain: A fixation-related fMRI study of syntactic surprisal in reading. Neuroimage. 2016;132:293–300. 10.1016/j.neuroimage.2016.02.050 26908322

[8] Martin AE, Doumas LAA. A mechanism for the cortical computation of hierarchical linguistic structure. PLoS Biol. 2017;15(3):e2000663 10.1371/journal.pbio.2000663 28253256 PMC5333798

[9] Frank SL, Bod R. Insensitivity of the human sentence-processing system to hierarchical structure. Psychol Sci. 2011;22(6):829–34. 10.1177/0956797611409589 21586764

[10] Frank SL, Otten LJ, Galli G, Vigliocco G. The ERP response to the amount of information conveyed by words in sentences. Brain and Language. 2015;140(0):1–11. 10.1016/j.bandl.2014.10.006. 25461915

[11] Brennan JR, Stabler EP, Van Wagenen SE, Luh WM, Hale JT. Abstract linguistic structure correlates with temporal activity during naturalistic comprehension. Brain Lang. 2016;157–158:81–94. 10.1016/j.bandl.2016.04.008 PMC4893969 27208858

[12] Chomsky N. Aspects of the Theory of Syntax. MIT press; 1965.

[13] Joshi A. How much context-sensitivity is required to provide reasonable structural descriptions: Tree adjoining grammars In: Dowty D, Karttunen L, Zwicky A, editors. Natural language parsing: Psychological, computational, and theoretical perspectives. Cambridge: Cambridge University Press; 1985 p. 206–250.

[14] Phillips C. The real-time status of island phenomena. Language. 2006;82(4):795–823. 10.1353/lan.2006.0217

[15] Sanford A, Sturt P. Depth of processing in language comprehension: not noticing the evidence. Trends Cogn Sci. 2002;6(9):382 10.1016/S1364-6613(02)01958-7 12200180

[16] Ferreira F, Bailey KGD, Ferraro V. Good-Enough Representations in Language Comprehension. Current Directions in Psychological Science. 2002;11(1):11–15. 10.1111/1467-8721.00158

[17] Townsend DJ, Bever TG. In: Sentence Comprehension: The Integration of Habits and Rules. Cambridge, MA: MIT Press; 2001.

[18] Marslen-Wilson W. Linguistic structure and speech shadowing at very short latencies. Nature. 1973;244(5417):522–3. 10.1038/244522a0 4621131

[19] Tanenhaus M, Spivey-Knowlton M, Eberhard K, Sedivy J. Integration of visual and linguistic information in spoken language comprehension. Science. 1995;268(5217):1632–1634. 10.1126/science.7777863 7777863

[20] Bever TG. The cognitive basis for linguistic structure In: Hayes JR, editor. Cognition and the development of language. New York: Wiley; 1970.

[21] Christiansen MH, Chater N. The Now-or-Never bottleneck: A fundamental constraint on language. Behav Brain Sci. 2016;39:e62 10.1017/S0140525X1500031X 25869618

[22] McDonald SA, Shillcock RC. Eye movements reveal the on-line computation of lexical probabilities during reading. Psychol Sci. 2003;14(6):648–52. 10.1046/j.0956-7976.2003.psci_1480.x 14629701

[23] Willems RM, Frank SL, Nijhof AD, Hagoort P, van den Bosch A. Prediction During Natural Language Comprehension. Cereb Cortex. 2015; 10.1093/cercor/bhv075 25903464

[24] Lopopolo A, Frank SL, van den Bosch A, Willems RM. Using stochastic language models (SLM) to map lexical, syntactic, and phonological information processing in the brain. PLoS One. 2017;12(5):e0177794 10.1371/journal.pone.0177794 28542396 PMC5436813

[25] Kutas M, Hillyard SA. Reading senseless sentences: Brain potentials reflect semantic incongruity. Science. 1980;207:203–205. 10.1126/science.7350657 7350657

[26] Kutas M, Hillyard SA. Brain Potentials during Reading Reflect Word Expectancy and Semantic Association. Nature. 1984;307(5947):161–162. 10.1038/307161a0 6690995

[27] Brennan JR, Pylkkänen L. MEG Evidence for Incremental Sentence Composition in the Anterior Temporal Lobe. Cognitive Science. 2017;41(S6):1515–1531. 10.1111/cogs.12445 27813182

[28] Hagoort P, Hald L, Bastiaansen M, Petersson KM. Integration of Word Meaning and World Knowledge in Language Comprehension. Science. 2004;304(5669):438–441. 10.1126/science.1095455 15031438

[29] Hale JT, Dyer C, Kuncoro A, Brennan JR. Finding syntax in human encephalography with beam search. In: Proceedings of the 56th Annual Meeting of the Association for Computational Linguistics (Volume 1: Long Papers). Association for Computational Linguistics; 2018. p. 2727–2736.

[30] Klein D, Manning CD. Accurate unlexicalized parsing. In: Proceedings of the 41st Annual Meeting on Association for Computational Linguistics-Volume 1. Association for Computational Linguistics; 2003. p. 423–430.

[31] Hale JT. A Probabilistic Earley Parser As a Psycholinguistic Model. In: Proceedings of the Second Meeting of the North American Chapter of the Association for Computational Linguistics on Language Technologies. NAACL’01. Stroudsburg, PA, USA: Association for Computational Linguistics; 2001. p. 1–8. Available from: 10.3115/1073336.1073357.

[32] Roark B, Bachrach A, Cardenas C, Pallier C. Deriving lexical and syntactic expectation-based measures for psycholinguistic modeling via incremental top-down parsing. In: Proceedings of the Conference on Empirical Methods in Natural Language Processing (EMNLP); 2009. p. 324–333.

[33] Neville H, Nicol JL, Barss A, Forster KI, Garrett MF. Syntactically Based Sentence Processing Classes: Evidence from Event-Related Brain Potentials. Journal of Cognitive Neuroscience. 1991;3(2):151–165. 10.1162/jocn.1991.3.2.151 23972090

[34] Lau EF, Stroud C, Plesch S, Phillips C. The role of structural prediction in rapid syntactic analysis. Brain and Language. 2006;98(1):74–88. 10.1016/j.bandl.2006.02.003 16620944

[35] Dikker S, Rabagliati H, Pylkkänen L. Sensitivity to syntax in visual cortex. Cognition. 2009;110(3):293–321. 10.1016/j.cognition.2008.09.008 19121826 PMC2709501

[36] Nieuwland M, Politzer-Ahles S, Heyselaar E, Segaert K, Darley E, Kazanina N, et al Large-scale replication study reveals a limit on probabilistic prediction in language comprehension. eLife. 2018 10.7554/eLife.33468 29631695 PMC5896878

[37] Roark B, Sproat R, Allauzen C, Riley M, Sorensen J, Tai T. The OpenGrm open-source finite-state grammar software libraries. In: Proceedings of the ACL 2012 System Demonstrations; 2012. p. 61–66.

[38] Elman JL. Finding Structure in Time. Cognitive Science. 1990;14(2):179–211. 10.1207/s15516709cog1402_1

[39] Sundermeyer M, Schlüter R, Ney H. rwthlm—The RWTH Aachen University Neural Network Language Modeling Toolkit. In: Proceedings of Interspeech; 2014.

[40] Frank SL. Uncertainty reduction as a measure of cognitive load in sentence comprehension. Top Cogn Sci. 2013;5(3):475–94. 10.1111/tops.12025 23681508

[41] Hale JT. Automaton Theories of Human Sentence Comprehension. CSLI Publications; 2014.

[42] Hale JT. Information-theoretical Complexity Metrics. Language and Linguistics Compass. 2016;10(9):397–412. 10.1111/lnc3.12196

[43] Luong MT, Frank MC, Johnson M. Parsing entire discourses as very long strings: Capturing topic continuity in grounded language learning. In: Transactions of the Association for Computational Linguistics (TACL13); 2013.

[44] Stolcke A. An Efficient Probabilistic Context-Free Parsing Algorithm that Computes Prefix Probabilities. Computational Linguistics. 1995;21(2):165–201.

[45] Levy R. Expectation-based syntactic comprehension. Cognition. 2008;106(3):1126–1177. 10.1016/j.cognition.2007.05.006 17662975

[46] Frank R, Mathis D, Badecker W. The Acquisition of Anaphora by Simple Recurrent Networks. Language Acquisition. 2013;20(3):181–227. 10.1080/10489223.2013.796950

[47] Linzen T, Dupoux E, Goldberg Y. Assessing the ability of LSTMs to learn syntax-sensitive dependencies. Transactions of the Association for Computational Linguistics. 2016;4:521–535.

[48] Gers FA, Schmidhuber E. LSTM recurrent networks learn simple context-free and context-sensitive languages. IEEE Transactions on Neural Networks. 2001;12(6):1333–1340. 10.1109/72.963769 18249962

[49] Blevins T, Levy O, Zettlemoyer L. Deep RNNs Encode Soft Hierarchical Syntax. In: Proceedings of the 56th Annual Meeting of the Association for Computational Linguistics (Volume 2: Short Papers). Association for Computational Linguistics; 2018. p. 14–19. Available from: http://aclweb.org/anthology/P18-2003.

[50] Dyer C, Kuncoro A, Ballesteros M, Smith NA. Recurrent Neural Network Grammars. In: Proceedings of the 2016 Conference of the North American Chapter of the Association for Computational Linguistics: Human Language Technologies. Association for Computational Linguistics; 2016. p. 199–209.

[51] Fine AB, Jaeger TF, Farmer TA, Qian T. Rapid Expectation Adaptation during Syntactic Comprehension. PLoS One. 2013;8(10):e77661 10.1371/journal.pone.0077661 24204909 PMC3813674

[52] Jelinek F.Statistical Methods for Speech Recognition. Cambridge, MA: MIT Press; 1998.

[53] Oostenveld R, Fries P, Maris E, Schoffelen JM. FieldTrip: Open source software for advanced analysis of MEG, EEG, and invasive electrophysiological data. Comput Intell Neurosci. 2011;2011:156869 10.1155/2011/156869 21253357 PMC3021840

[54] Jung TP, Makeig S, Westerfield M, Townsend J, Courchesne E, Sejnowski TJ. Removal of eye activity artifacts from visual event-related potentials in normal and clinical subjects. Clin Neurophysiol. 2000;111(10):1745–58. 10.1016/S1388-2457(00)00386-2 11018488

[55] Perrin F, Pernier J, Bertrand O, Giard MH, Echallier JF. Mapping of scalp potentials by surface spline interpolation. Electroencephalogr Clin Neurophysiol. 1987;66(1):75–81. 10.1016/0013-4694(87)90141-6 2431869

[56] Balota DA, Yap MJ, Cortese MJ, Hutchinson KI, Kessler B, Loftis B, et al The English Lexicon Project. Behavior Research Methods. 2007;39:445–459. 10.3758/BF03193014 17958156

[57] Maris E, Oostenveld R. Nonparametric Statistical Testing of EEG- and MEG-data. Journal of Neuroscience Methods. 2007;164(1):177–190. 10.1016/j.jneumeth.2007.03.024 17517438

[58] Vul E, Harris C, Winkielman P, Pashler H. Puzzlingly High Correlations in fMRI Studies of Emotion, Personality, and Social Cognition. Perspect Psychol Sci. 2009;4(3):274–90. 10.1111/j.1745-6924.2009.01125.x 26158964

[59] Bürkner PC. brms: An R Package for Bayesian Multilevel Models Using Stan. Journal of Statistical Software. 2017;80(1):1–28.

[60] Carpenter B, Gelman A, Hoffman M, Lee D, Goodrich B, Betancourt M, et al Stan: A Probabilistic Programming Language. Journal of Statistical Software, Articles. 2017;76(1):1–32. 10.18637/jss.v076.i01 PMC9788645 36568334

[61] Nicenboim B, Vasishth S. Statistical methods for linguistic research: Foundational Ideas—Part II. Language and Linguistics Compass. 2016;10(11):591–613. 10.1111/lnc3.12207

[62] Vehtari A, Gelman A, Gabry J. Practical Bayesian model evaluation using leave-one-out cross-validation and WAIC. Statistics and Computing. 2017;27(5):1413–1432. 10.1007/s11222-016-9696-4

[63] Smith NJ, Kutas M. Regression-based estimation of ERP waveforms: I. The rERP framework. Psychophysiology. 2015;52(2):157–68. 10.1111/psyp.12317 25141770 PMC5308234

[64] Kutas M, Federmeier KD. Thirty years and counting: finding meaning in the N400 component of the event-related brain potential (ERP). Annu Rev Psychol. 2011;62:621–47. 10.1146/annurev.psych.093008.131123 20809790 PMC4052444

[65] Alday PM, Schlesewsky M, Bornkessel-Schlesewsky I. Electrophysiology Reveals the Neural Dynamics of Naturalistic Auditory Language Processing: event-Related Potentials Reflect Continuous Model Updates. eNeuro. 2017;4(6). 10.1523/ENEURO.0311-16.2017 29379867 PMC5779117

[66] Friederici AD, Pfeifer E, Hahne A. Event-related brain potentials during natural speech processing: Effects of semantic, morphological and syntactic violations. Cognitive Brain Research. 1993;1(3):183–192. 10.1016/0926-6410(93)90026-2 8257874

[67] Bradley DC. Computational Distinctions of Vocabulary Type. Indiana University Linguistics Club; 1983.

[68] Molinaro N, Barber HA, Carreiras M. Grammatical agreement processing in reading: ERP findings and future directions. Cortex. 2011;47(8):908–30. 10.1016/j.cortex.2011.02.019 21458791

[69] Tanner D. On the left anterior negativity (LAN) in electrophysiological studies of morphosyntactic agreement: a commentary on “grammatical agreement processing in reading: ERP findings and future directions” by Molinaro et al., 2014. Cortex. 2015;66:149–55. 10.1016/j.cortex.2014.04.007 24928489

[70] Molinaro N, Barber HA, Caffarra S, Carreiras M. On the left anterior negativity (LAN): The case of morphosyntactic agreement: a reply to Tanner et al. Cortex. 2015;66:156–9. 10.1016/j.cortex.2014.06.009 25017646

[71] Steinhauer K, Drury JE. On the early left-anterior negativity (ELAN) in syntax studies. Brain and Language. 2012;120(2):135–162. 10.1016/j.bandl.2011.07.001 21924483

[72] Gulordava K, Bojanowski P, Grave E, Linzen T, Baroni M. Colorless green recurrent networks dream hierarchically. In: Proceedings of NAACL HLT 2018; 2018 Available from: http://arxiv.org/abs/1803.11138.

[73] Dikker S, Rabagliati H, Farmer T, Pylkkänen L. Early occipital sensitivity to syntactic category is based on form typicality. Psychological Science. 2010;21(5):629–634. 10.1177/0956797610367751 20483838

[74] Dikker S, Pylkkänen L. Predicting language: MEG evidence for lexical preactivation. Brain Lang. 2012; 10.1016/j.bandl.2012.08.004 23040469

