# Supplementary material for: Hierarchical structure guides rapid linguistic predictions during naturalistic listening
Source: PLoS One. 2019 Jan 16;14(1):e0207741. doi: 10.1371/journal.pone.0207741 (PMC6334990; doi:10.1371/journal.pone.0207741)
Supplement: S1 File — Supplemental materials include further examples of model output, figures for all whole-head results, and analyses that include participants who did not meet behavioral criteria. (PDF) [file pone.0207741.s001.pdf]

Supplemental Material for "Hierarchical structure guides rapid linguistic predictions during naturalistic listening"

Jonathan R. Brennan*, *University of Michigan*
John T. Hale, *Cornell University*

November 2, 2018

*Corresponding Author: jobrenn@umich.edu

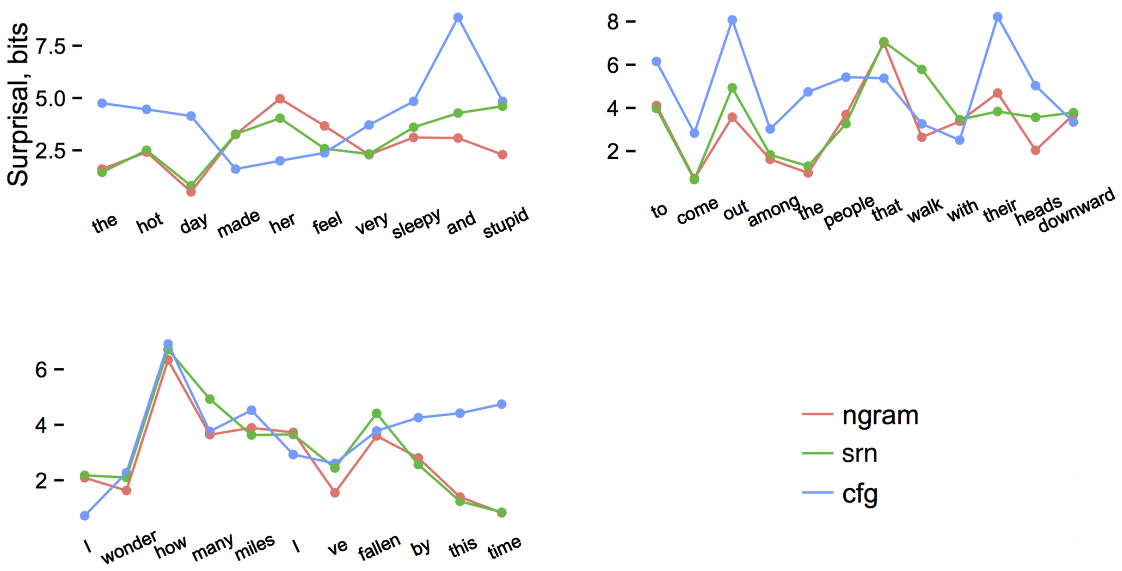

Figure S1: **Example sentences** showing word-by-word suprisal estimates from each of the three models.

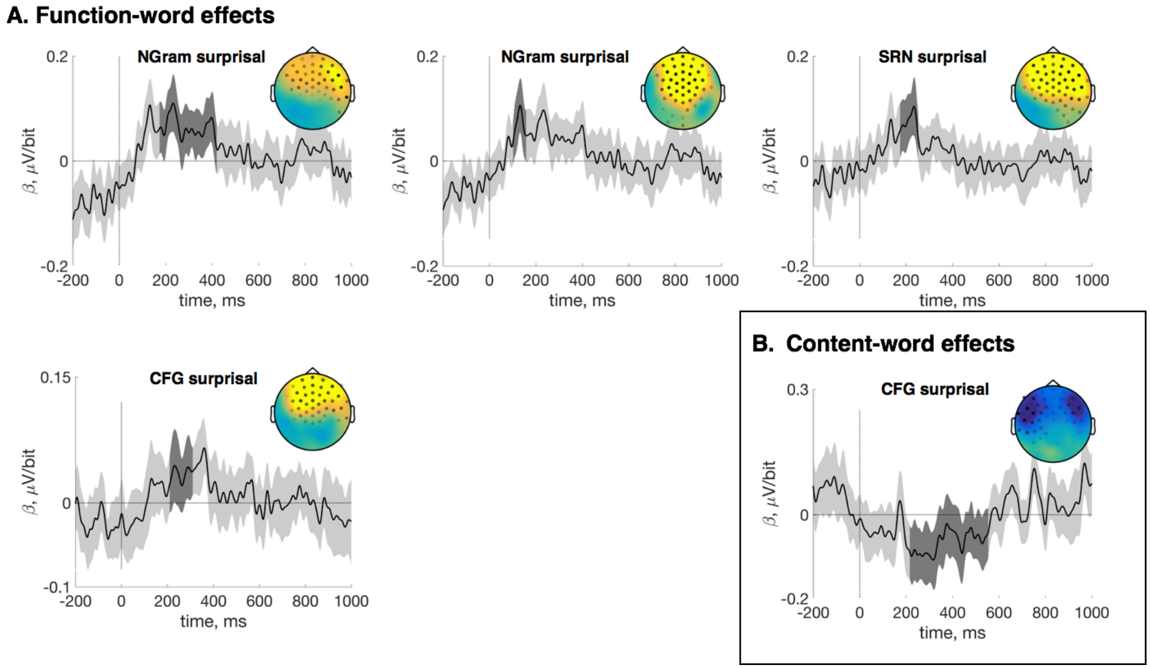

Figure S2: **All significant whole-head effects** for surprisal terms from the whole-head analysis, divided by whether they are fit against function-word data (A), or content-word data (B). Black lines represent the time-series of regression fits ($\beta \pm CI_{95}$) for the indicated surprisal term. Dark grey shading indicates significant time-points and inset shows significant channels and coefficient averages across significant time-points. It appears as if the various function-word effects have significant temporal and electrode overlap. Model comparisons (Tables 3 and S1) indicate that *Ngram* and *SRN* surprisals significantly improve model fits over *CFG* for these electrodes and time-windows. Despite the specificity to function-words in this whole-head analysis, such improvements do not interact with function-vs-content word-class when such an interaction is explicitly tested.

	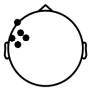 216 – 554 ms	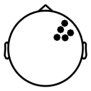 216 – 554 ms	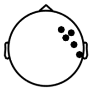 174 – 420 ms	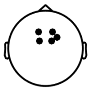 102 – 158 ms	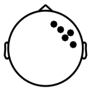 174 – 252 ms	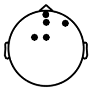 210 – 310 ms
baseline: NGRAM + RNN + controls						
CFG:WC + CFG > baseline	**29.59 (11.66)**	**23.62 (10.37)**	**24.18 (10.42)**	6.20 (6.28)	7.45 (6.66)	**29.16 (11.31)**
CFG:WC > CFG + baseline	**22.54 (9.97)**	**23.41 (9.91)**	**21.00 (9.57)**	7.16 (6.02)	2.16 (4.15)	**29.33 (11.14)**
CFG > baseline	7.05 (6.26)	0.21 (3.20)	3.18 (4.39)	-0.95 (1.83)	5.28 (5.27)	-0.17 (2.30)
baseline: CFG + controls						
NGRAM:WC + NGRAM > baseline	13.08 (8.55)	**28.94 (11.41)**	**52.80 (15.33)**	12.68 (8.05)	**35.16 (12.80)**	**21.20 (10.09)**
NGRAM:WC > NGRAM + baseline	-2.24 (0.81)	3.26 (3.86)	5.65 (5.78)	3.29 (4.27)	6.23 (6.04)	-1.93 (0.65)
NGRAM > baseline	15.31 (8.52)	**25.68 (10.72)**	**47.15 (14.03)**	9.39 (6.95)	**28.93 (11.16)**	**23.14 (10.05)**
baseline: CFG + controls						
RNN:WC + RNN > baseline	9.46 (7.69)	13.14 (8.20)	**42.97 (13.86)**	3.10 (5.64)	**41.74 (13.73)**	16.40 (9.11)
RNN:WC > RNN + baseline	-1.84 (0.31)	-1.36 (1.85)	4.08 (4.87)	-2.52 (0.31)	5.87 (6.16)	-1.99 (0.75)
RNN > baseline	11.30 (7.71)	14.50 (7.98)	**38.89 (12.83)**	5.62 (5.67)	**35.86 (12.11)**	**18.39 (9.07)**
baseline: NGRAM + controls						
RNN:WC + RNN > baseline	-2.77 (0.70)	-3.07 (2.65)	-3.33 (1.92)	2.48 (5.47)	0.94 (4.86)	-4.24 (0.38)
RNN:WC > RNN + baseline	-1.46 (0.34)	-1.67 (0.74)	-1.69 (1.57)	4.47 (5.48)	-0.38 (2.32)	-2.36 (0.37)
RNN > baseline	-1.31 (0.69)	-1.40 (2.52)	-1.65 (1.12)	-1.98 (0.74)	1.31 (4.17)	-1.88 (0.26)

Table S1: **Auxilliary model comparison results** including 8 participants who did not meet the pre-specified behavioral criterion (total N = 41). The table shows WAIC difference scores ($\pm$ standard error) for changes in model fit across six ROIs (columns). Each set of rows tests a different statistical question using step-wise model comparison. Terms that are being evaluated are indicated to the left of ">"; interactions with word-class are indicated with ":WC". For each, the baseline model includes all control covariates along with the indicated surprisal term(s) and as any interactions between word-class and those surprisal terms. The values are scaled so that positive numbers represent improvements for the larger model, while negative numbers indicate that the added complexity of the larger model is not justified. Bold-face indicates WAIC improvements that are more than two standard errors from zero.

		**Rhat**		**R2**		**AR(1)**	
		min	*max*	*min*	*max*	*min*	*max*
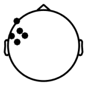	216–554 ms	0.999	1.004	0.0004	0.0043	0.187	0.188
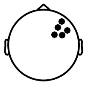	216–554 ms	0.999	1.003	0.0005	0.0043	0.171	0.171
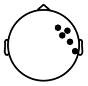	174–420 ms	0.999	1.003	0.0005	0.0056	0.144	0.145
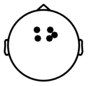	102–158 ms	0.999	1.016	0.0005	0.0044	0.068	0.068
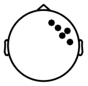	174–252 ms	0.999	1.001	0.0005	0.0056	0.081	0.081
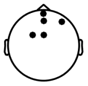	210–310 ms	0.999	1.007	0.0006	0.0069	0.052	0.053

Table S2: **Model goodness-of-fit** summaries from the set of models fit against each of the 6 ROIs examined in the model-comparison analysis. *RHat* summarizes the concordance between chains used in the Monte-Carlo estimation procedure and should be below 1.1. R^2 is a Bayesian formulation of the proportion of variance explained in the *single-trial EEG data* (`bayes_R2()` function from the *rstantools* package). *AR(1)* indicates that the first-order auto-correlation of the residuals is low.
